# Transcriptome sequencing of hepatocellular carcinoma uncovers multiple types of dysregulated ncRNAs

**DOI:** 10.3389/fonc.2022.927524

**Published:** 2022-09-05

**Authors:** Li Zhang, Chunmei Wang, Xiaojie Lu, Xiao Xu, Tieliu Shi, Jinlian Chen

**Affiliations:** ^1^ Department of Gastroenterology, Affiliated Sixth People’s Hospital South Campus of Shanghai Jiaotong University, Shanghai, China; ^2^ Center for Bioinformatics and Computational Biology, The Institute of Biomedical Sciences, School of Life Sciences, East China Normal University, Shanghai, China; ^3^ Department of Gastroenterology, Affiliated Fengxian Hospital of Southern Medical University, Shanghai, China; ^4^ The First Affiliated Hospital, Zhejiang University, Hangzhou, China; ^5^ Shanghai East Hospital, Tongji University School of Medicine, Shanghai, China

**Keywords:** transcriptome profiling, noncoding RNAs, competing endogenous RNA networks, miRNA sponge, tumorigenesis

## Abstract

Transcriptome profiling of hepatocellular carcinoma (HCC) by next-generation sequencing (NGS) technology has been broadly performed by previous studies, which facilitate our understanding of the molecular mechanisms of HCC formation, progression, and metastasis. However, few studies jointly analyze multiple types of noncoding RNAs (ncRNAs), including long noncoding RNAs (lncRNAs), circular RNAs (circRNAs), and micro-RNAs (miRNAs), and further uncover their implications in HCC. In this study, we observed that the circRNA cZRANB1 and lncRNA DUXAP10 were not only significantly upregulated in tumor tissues, but also higher expressed in blood exosomes of HCC as compared with healthy donors. From the analysis of subclass-associated dysregulated ncRNAs, we observed that DLX6-AS1, an antisense RNA of DLX6, and the sense gene DLX6 were highly expressed in S1, a subclass with a more invasive/disseminative phenotype. High correlation between DLX6-AS1 and DLX6 suggested that DLX6-AS1 may function *via* promoting the transcription of DLX6. Integrative analysis uncovers circRNA–miRNA, lncRNA–miRNA, and competing endogenous RNA networks (ceRNAs). Specifically, cZRANB1, LINC00501, CTD-2008L17.2, and SLC7A11-AS1 may function as ceRNAs that regulate mRNAs by competing the shared miRNAs. Further prognostic analysis demonstrated that the dysregulated ncRNAs had the potential to predict HCC patients’ overall survival. In summary, we identified some novel circRNAs and miRNAs, and dysregulated ncRNAs that could participate in HCC tumorigenesis and progression by inducing transcription of their neighboring genes, increasing their derived miRNAs, or acting as miRNA sponges. Moreover, our systematic analysis provides not only rich data resources for related researchers, but also new insights into the molecular basis of how different ncRNAs coordinately or antagonistically participate in the pathogenesis process of diseases.

## Introduction

Hepatocellular carcinoma (HCC) is one of the most common malignancies and the second leading cause of cancer deaths worldwide (1). It is supposed that newly diagnosed HCC cases each year will be more than 1 million worldwide in 2025 (2, 3). Moreover, both morbidity and mortality of HCC in China are higher than other countries. Overall, the delayed diagnosis of a majority of HCC patients prohibits surgery or other effective radical treatments, resulting in poor prognosis, which makes the 5-year survival rate less than 15% (4). Currently, the widely used clinical biomarker for HCC diagnosis is alpha fetoprotein (AFP), while its sensitivity is only about 60% (5). The available targeted drug recommended by definitive guides in clinical practice (6) for advanced HCC is sorafenib, which, however, is limited in improving the overall survival (7, 8). Besides sorafenib, other targeted drugs, such as sunitinib (9), brivanib (10), and everolimus (11), were tested in clinical trials, but all failed in the third phase (4).

The latest developments in next-generation sequencing (NGS) technologies have profiled mutational spectrums, and deregulated expression and epigenetic changes of several cancers by TCGA studies (12). Whole genome or exome sequencing of HCC has identified some recurrently mutated genes, such as TERT promoter (54%–60%), p53 (12%–48%), β-catenin (11%–37%), and Axin (5%–15%) (13). Transcriptome sequencing, including RNA sequencing and small RNA sequencing, of cancers shows remarkable potential to identify both novel markers and uncharacterized aspects of tumor biology, particularly some non-coding RNAs. Studies about microRNAs (miRNAs) show that miRNAs are closely related to HCC tumorigenesis, development, and metastasis (14, 15). For example, the downregulation of miR-28-5p is related to HCC metastasis, recurrence, and poor prognosis (16). MiR-188-5p can inhibit the proliferation and metastasis of HCC by targeting FGF5 (17). Decreased expression of miRNA-122 has been frequently detected in hepatitis B virus (HBV)-related HCCs, and re-expressing of miRNA-122 conversely inhibits HCC progression (18). Moreover, long non-coding RNAs (lncRNAs), which are generally unable to encode proteins, are also involved in tumor formation, development, or metastasis. Suppression of lncRNA Hotair can significantly inhibit the expression of miR-218 and induce cell cycle arrest in G1 phase and thereby inhibit HCC progression (19). Overexpression of lncRNA HULC in liver cancer promotes HCC proliferation by downregulating tumor suppressor gene p18 (20). In addition to lncRNAs and miRNAs, circular RNAs (circRNAs), produced by non-canonical splicing events that join a splice donor to an upstream splice acceptor, are also abundant and conserved in both normal and tumor cells (21). Numerous studies show that circRNAs can act as key regulators in cancer by regulating transcription or post-transcription of driver genes (22–24).

**Figure 1:**
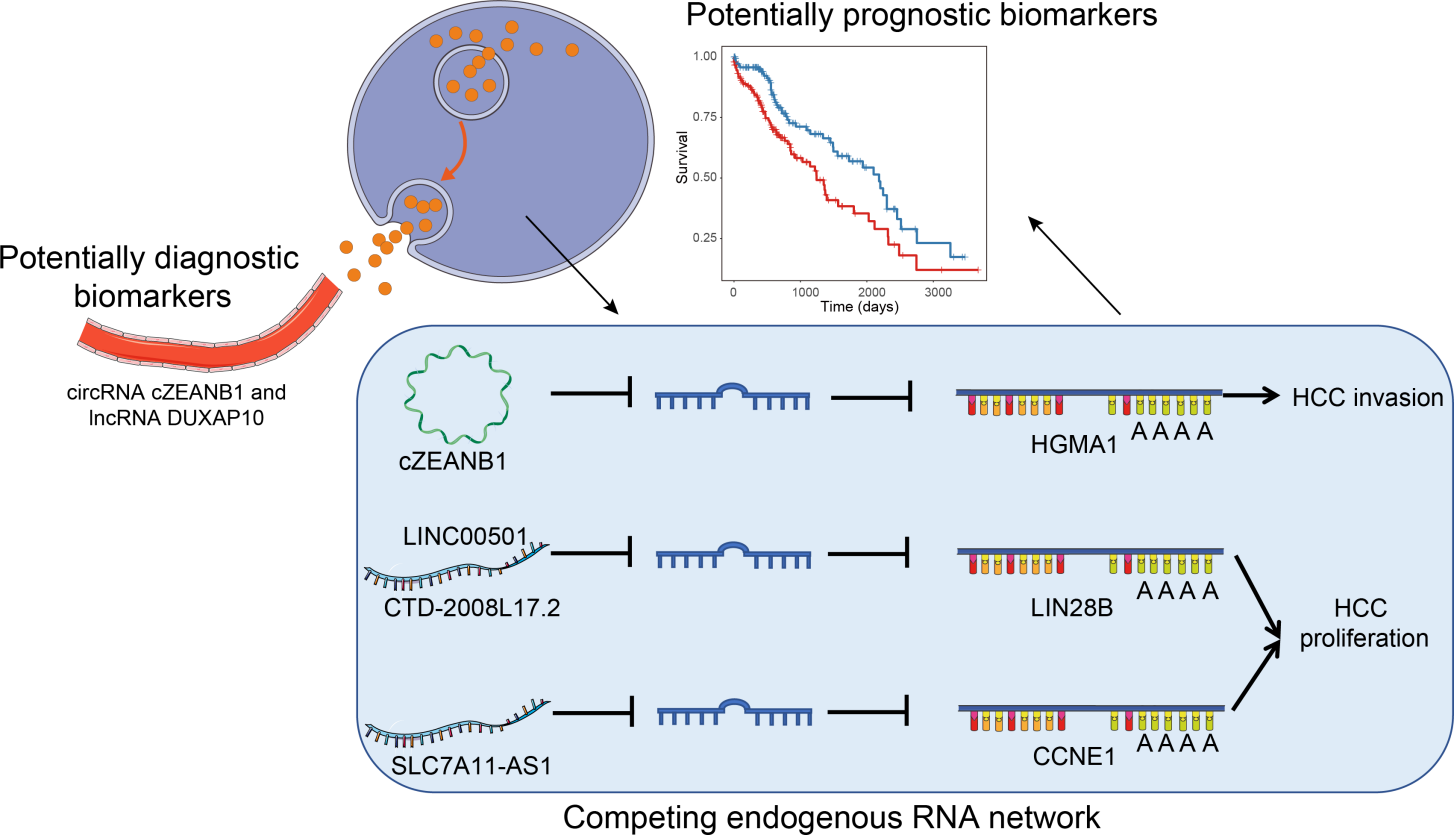
The overall major findings of this study.

To comprehensively profile multiple types of ncRNAs in HCC and uncover their roles in HCC tumorigenesis or progression, we performed RNA sequencing in parallel with small RNA sequencing of eight paired HCC and matched pare-cancerous tissues. From the systematic comparisons, we characterized tumorigenesis- and subclass-associated ncRNAs, and predicted their potential biological function and lncRNA/circRNA–miRNA–mRNA interactions (**Figure 1**), which highlighted some key functional ncRNAs at the post-transcriptional level.

## Results

### Transcriptome profiling of multiple types of RNAs in HCC and matched para-cancerous tissues

**Figure 2:**
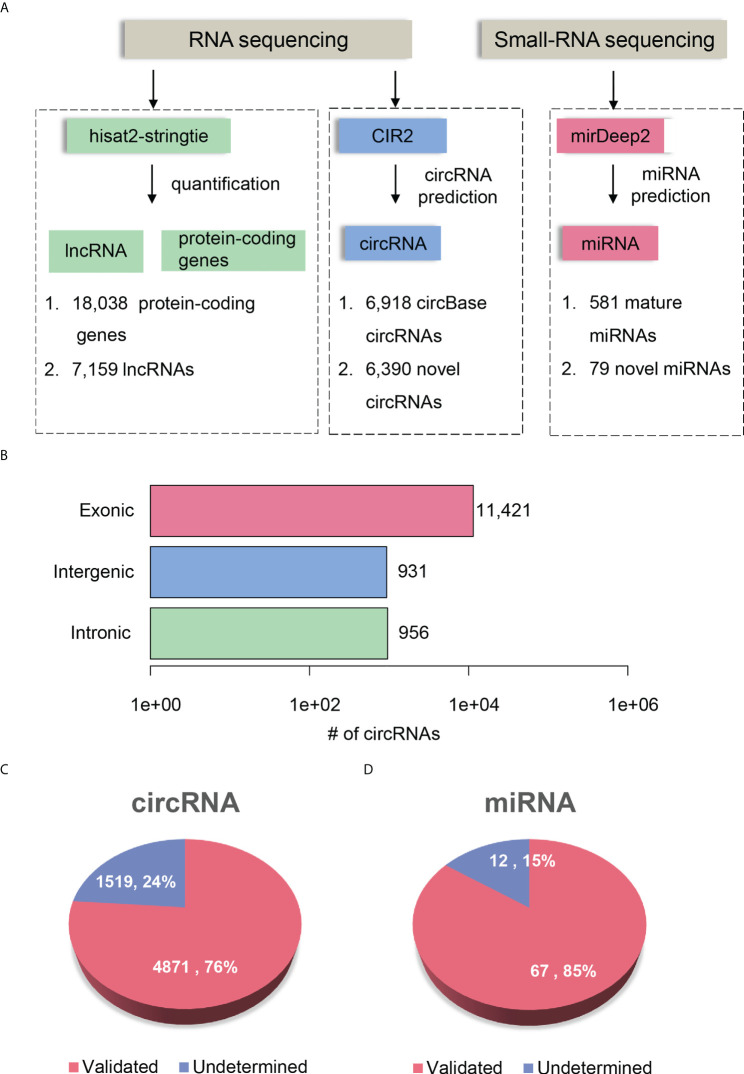
Transcriptome profiling of non-coding RNAs by RNA-seq in HCC. **(A)** The workflow for ncRNA detection and quantification. The circRNA and miRNA detection were performed by CIRI2 and miRDeep2, respectively. **(B)** The number of circRNAs originated from different genomic regions, including exonic, intronic, and intergenic regions. The novel circRNAs and miRNAs are displayed in pie charts of **(C, D)**. The pink and blue parts represent the validated and undetermined circRNAs/miRNAs by the independent dataset, respectively.

To comprehensively delineate the transcriptome of HCC and matched para-cancerous tissues, we performed small RNA sequencing and rRNA depletion-based total RNA sequencing on eight pairs of HCC and matched para-cancerous tissues, respectively. The workflow analyzing the small RNA and RNA sequencing datasets is illustrated in **Figure 2A**. We detected 581 mature miRNAs annotated by miRBase (25) (version 20) and 79 novel miRNAs annotated by miRDeep2 (26) with small RNA sequencing (**Supplementary Table 1**).

RNA sequencing datasets were processed by the reference-based (GENCODE (27) version 19) gene expression quantification pipeline and circular RNA (circRNA) prediction pipeline, respectively. We identified a total of 25,197 genes, which consists of 18,038 protein-coding genes and 7,159 lncRNAs (read count >5 in more than four samples). In addition, RNA sequencing based on rRNA-depleted RNA library made it feasible to detect and quantify circRNAs. The circRNA prediction pipeline for the RNA sequencing data identified 13,308 circRNAs, 6,918 of which were annotated by circBase (28) (**Supplementary Table 2**). To annotate the origin of circRNAs, we determined whether the splice sites of circRNAs have been annotated according to the gene annotation from GENCODE. About 85.82%, 7.0%, and 7.18% of the circRNAs originated from exonic, intergenic, and intronic regions, respectively (**Figure 2B**). Moreover, we observed 52.81% of genes produced more than two circRNA isoforms, which indicated that alternative circularization extensively existed in HCC and para-cancerous tissues.

To further investigate whether the novel circRNAs and miRNAs could be identified in an independent HCC cohort, we performed identical data analysis on the publicly available RNA-seq and miRNA-seq datasets of 20 paired HCC and para-cancerous tissues (29). For the novel ncRNAs, including circRNAs and miRNAs, 4,871 circRNAs (76%) and 67 miRNAs (85%) were identified in the independent HCC cohort (**Figures 2C, D**). The high reproducibility demonstrated that the circRNA and miRNA prediction pipelines were robust, and the novel ncRNAs were highly reliable.

### Identification and validation of dysregulated ncRNAs in HCC tumorigenesis

**Figure 3:**
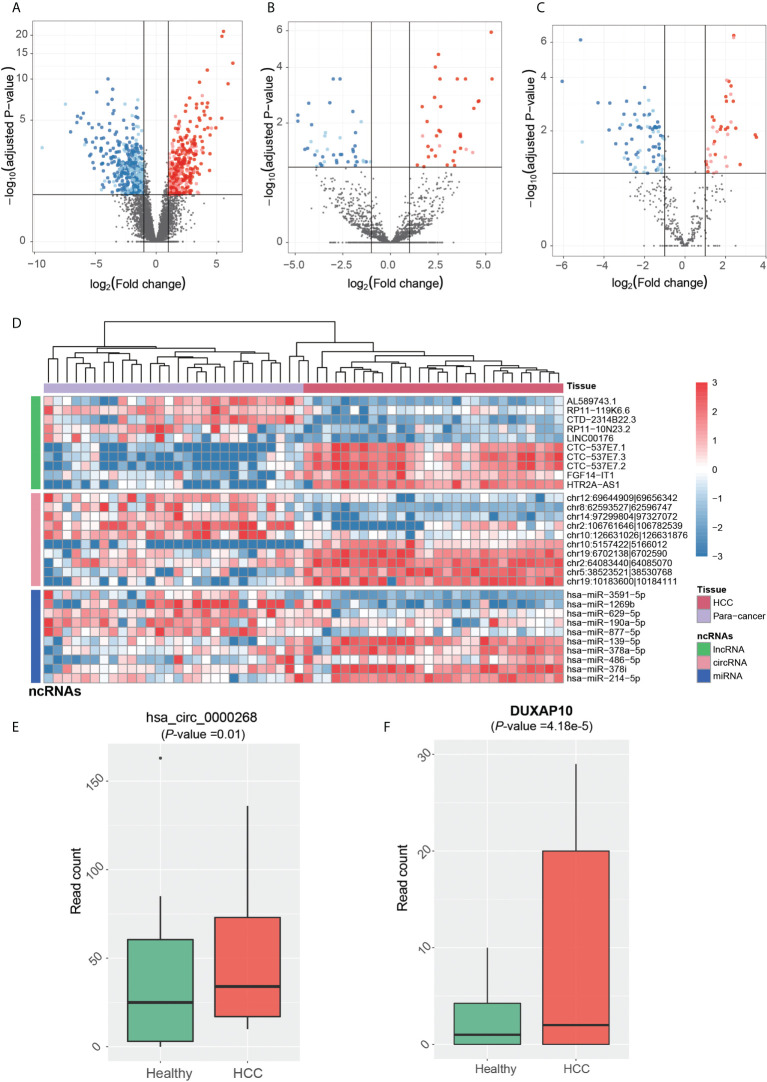
Tumorigenesis-associated dysregulated ncRNAs in HCC. The expression patterns of lncRNAs, circRNAs, and miRNAs are displayed in **(A-C)**. The red and blue points represent up- and downregulated ncRNAs in HCC. The solid points were also detected to be differentially expressed in the independent dataset. **(D)** The normalized expression levels of each of the top 10 ncRNAs by differential expression analysis, which are separated by three panels. HCC and para-cancerous samples were clustered by hierarchical clustering analysis, and are represented by the red and purple band on the top. The expression patterns of circRNA (hsa_circ_0000268) and lncRNA DUXAP10 in blood exosomes of HCC and healthy donors are displayed in **(E, F)**.

The dysregulated ncRNAs in HCC tumorigenesis should be aberrantly expressed between primary HCC and para-cancerous tissues. Prior to identifying the dysregulated ncRNAs in HCC tumorigenesis, the ncRNAs were quantified at count-based level to perform differential expression analysis. In total, we identified 844 lncRNAs, 80 circRNAs, and 114 miRNAs, which were differentially expressed between HCC and para-cancerous tissues (adjusted *p*-value <0.1 and fold-change >2 or < ^1^/_2_). The dysregulation of ~65% lncRNAs, ~76% circRNAs, and ~62% miRNAs was validated in the independent dataset (**Figures 3A–C**), including 20 novel circRNAs and one novel miRNA. Among the dysregulated ncRNAs validated by the independent dataset, PVT1, GAS5, DDX11-AS1, LINC-ROR, hsa-miR-483-5p, hsa-miR-139-5p, hsa-miR-150-5p, hsa-miR-195-5p, and hsa-miR-199a-5p were associated with HCC by previous studies (30–38).

We then conducted hierarchical clustering analysis to obtain the systematic comparison of ncRNA expression levels across different samples. Using each of the top 10 ncRNAs, HCC tissues were clustered into the same branch, and the para-cancerous tissues were clustered in the other branch, indicating that the ncRNAs could clearly distinguish cancerous from para-cancerous tissues (**Figure 3D**). To determine whether these ncRNAs were present in HCC blood exosomes, we investigated their expression patterns in the blood exosomes of 21 HCC and 32 healthy donors from the publicly available RNA-seq dataset (see *Materials and Methods*).

Notably, one upregulated circRNA hsa_circ_0000268 (cZRANB1) in HCC was also expressed in the exosome of HCC patients higher than the healthy donors (**Figure 3E**, *p*-value = 0.01). Moreover, the lncRNA DUXAP10 was also highly expressed in the exosome of HCC patients as compared with healthy controls (**Figure 3F**, *p*-value = 4.18e-5). In addition to potentially promoting HCC tumorigenesis, the two upregulated ncRNAs, especially the lncRNA DUXAP10, in the exosome of HCC patients may also be potential diagnostic biomarkers for HCC. The results suggested that the dysregulated ncRNAs in HCC may not only contribute to the tumorigenesis, but also have the potential to be diagnostic biomarkers for HCC.

### Identification of dysregulated ncRNAs in HCC subclasses

**Figure 4:**
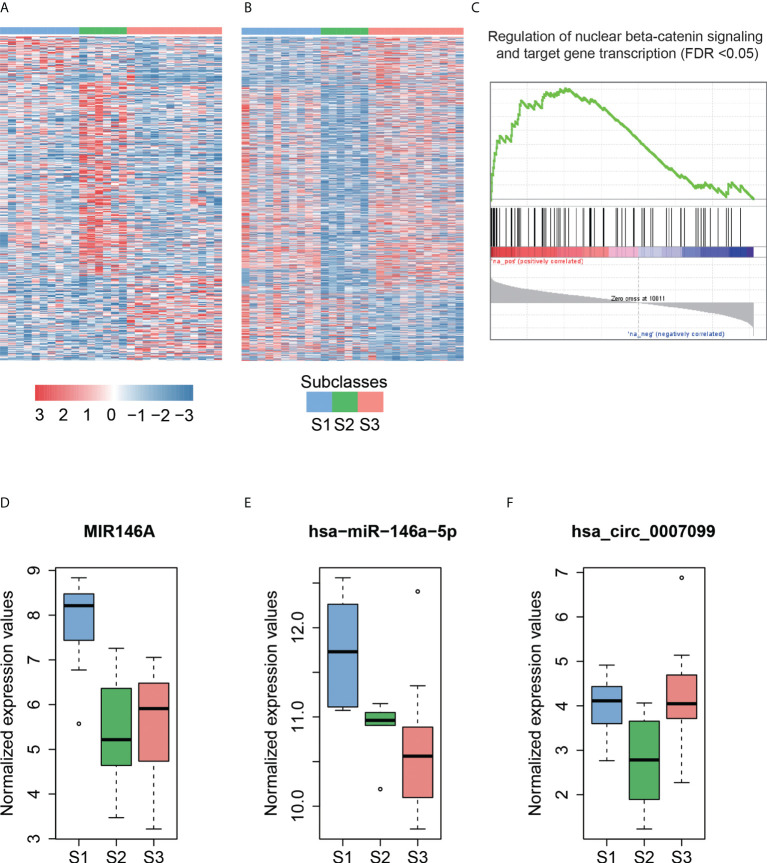
The subclass-associated dysregulated ncRNAs in HCC. The upregulated **(A)** and downregulated **(B)** ncRNAs in Hoshida subclasses. **(C)** Co-expression-based GSEA for DLX6-AS1. The highly correlated genes with DLX6-AS1 are significantly enriched in the gene set from regulation of nuclear beta-catenin signaling and target gene transcription. The expression patterns of MIR146A **(D)** and hsa-miR-146a-5p **(E)** in Hoshida subclasses. The miRNA host gene, MIR146A, and its derived miRNA, hsa-miR-146a-5p, are upregulated in the S1 subclass. **(F)** The hsa_circ_0007099 is downregulated in the S2 subclass.

Hoshida et al. (39) defined three robust HCC subclasses (termed S1, S2, and S3) based on a meta-analysis of gene expression profiles in datasets from eight independent patient cohorts. We classified 8 HCCs in this study and another 20 HCCs in the independent dataset into three subclasses (S1, S2, and S3) with 10, 6, and 12 cases based on the expression patterns of 619 signatures (*Materials and Methods*). We then performed differential expression analysis to identify dysregulated ncRNAs for each subclass (**Figures 4A, B**, adjusted *p*-value <0.25 and fold change >2 or < ^1^/_2_, *Materials and Methods*).

As described by Hoshida et al. (39), S1 is a subclass with a more invasive/disseminative phenotype. We observed that DLX6-AS1, an antisense RNA of DLX6, was highly expressed in S1. A previous study (40) found that DLX6-AS1 was highly expressed in lung adenocarcinoma, and knockdown of DLX6-AS1 could significantly decrease the mRNA and protein expression of DLX6. Accordingly, high correlation (Pearson correlation coefficient, PCC > 0.8) between DLX6-AS1 and DLX6 was observed in HCC and para-cancerous tissues, suggesting that DLX6-AS1 may cis-regulate DLX6 transcription in HCC. Co-expression-based GSEA revealed that DLX6-AS1 was co-expressed with genes involved in the regulation of nuclear beta-catenin signaling and target gene transcription (**Figure 4C**), such as SALL4, ZCCHC12, and BCL9, suggesting that DLX6-AS1 may participate in beta-catenin-induced gene transcription in HCC.

Notably, the lncRNA MIR146A and its derived miRNA hsa-miR-146a-5p were significantly upregulated in the S1 subclass (**Figures 4D, E**), indicating that MIR146A may function *via* promoting the transcription of their derived miRNAs. To our knowledge, most of the subclass-related dysregulated circRNAs have not been reported previously. Exceptionally, hsa_circ_0007099, which was downregulated in S2, was reported to be downregulated in gastric cancer (41) (**Figure 4F**). As characterized by Hoshida et al. (39), MYC and PI3K/Akt activations were the features of subclass S2, which may be inversely associated with hsa_circ_0007099. In summary, the subclass-associated dysregulated ncRNAs may participate in some signaling pathways specifically activated or inactivated in a certain subclass, and were strongly associated with some clinical characteristics.

### Identification of functional ncRNAs by constructing ceRNA network

From the systematic analysis above, we identified 1,598 dysregulated lncRNAs and 284 circRNAs; however, for most of them, their functionality remained unknown. To identify the functional ncRNAs responsible for HCC tumorigenesis or progression, we predicted the potential regulatory network involving lncRNAs, circRNAs, miRNAs, and mRNAs, namely, ceRNA (competing endogenous RNA) networks. As ceRNAs could regulate mRNAs by competing for the shared miRNAs, we firstly predicted the miRNA binding sites of the dysregulated ncRNAs using miRanda. Secondly, the predicted and experimentally validated mRNA–miRNA interactions were obtained from TargetScan (42) and miRTarBase (43) databases, respectively. With the threshold at −0.4 for the correlation coefficient between miRNA and target, we successfully predicted 152,881 lncRNA–miRNA pairs, 2,063 circRNA–miRNA pairs, and 8,056 mRNA–miRNA pairs, respectively.

**Figure 5:**
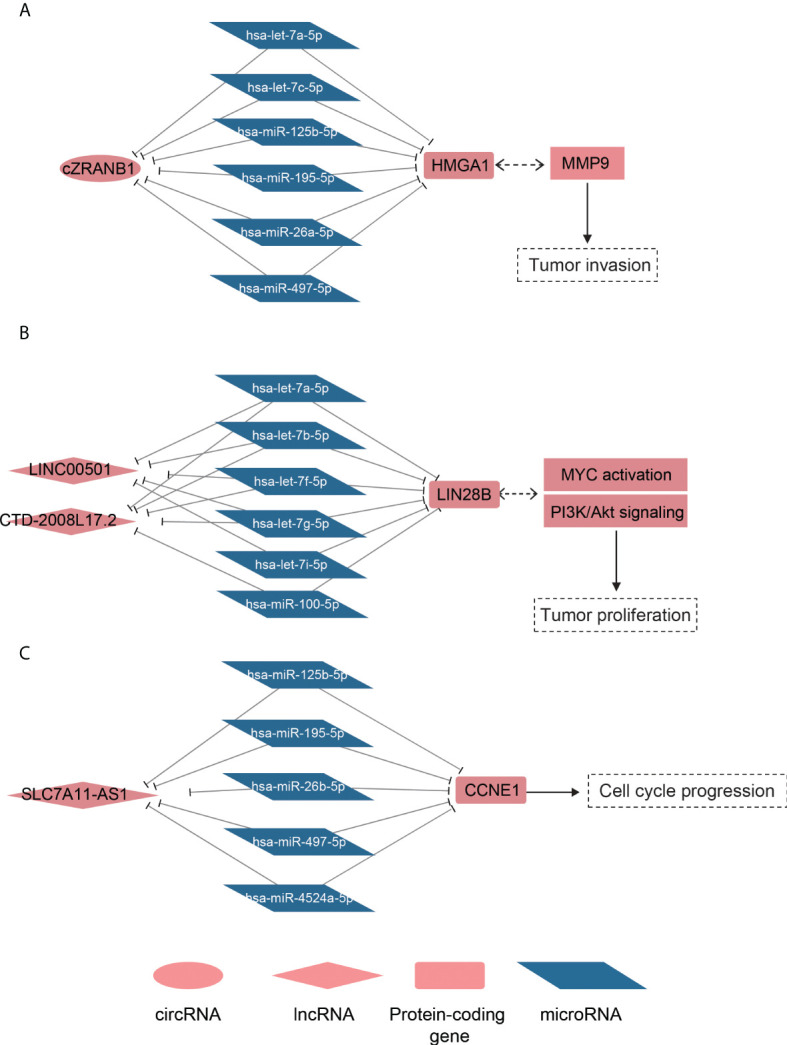
The ceRNA networks for dysregulated ncRNAs. The schematic diagrams for the cZRANB1, LINC00501/CTD-2008L17.2, and SLC7A11-AS1 are displayed in **(A-C)**. The round, rhombus, rectangle, and rhomboid symbols represent the circRNAs, lncRNAs, mRNAs, and microRNAs, respectively. The symbols filled with red or blue color represent up- or downregulated in HCC.

To model the ceRNA network, we performed the hypergeometric test combined with co-expression to predict the ncRNA–miRNA–mRNA regulatory relationships. Finally, we predicted 219 lncRNA–mRNA pairs and 31 circRNA–mRNA pairs (**Supplementary Table 3**). Notably, the circRNA cZRANB1 (hsa_circ_0000268), which was identified as a miRNA sponge by a previous study (44), shared five miRNAs, including hsa-let-7a-5p, hsa-let-7c-5p, hsa-miR-26a-5p, hsa-miR-125b-5p, hsa-miR-195-5p, and hsa-miR-497-5p, with HMGA1 (**Figure 5A**). In particular, cZEANB1 was upregulated in both HCC tissues and exosomes. HMGA1 has been found to associate with tumor invasion in several cancers (45–48), which was also observed in HCC based on its significantly positive correlation with MMP9 (PCC > 0.6). The results indicated that cZRANB1 may promote tumor invasion by competing for the miRNAs with HMGA1, thereby increasing the expression level of HMGA1.

For the lncRNA–mRNA pairs, we noticed that two lncRNAs, LINC00501 and CTD-2008L17.2, which were overexpressed in S2 subclass, may compete for miRNAs with LIN28B (**Figure 5B**). As described above, the S2 subclass was characterized by activation of MYC and the PI3k/Akt pathway, and the ceRNA network may be associated with these activations. In particular, the shared miRNAs belonged to the let-7 family, which function as tumor suppressor in several cancers (49, 50). Interestingly, LIN28B could also suppress the biogenesis of miRNAs including let-7 family miRNAs (51), indicating that LINC00501, CTD-2008L17.2, let-7 family miRNAs, and LIN28B constituted a complex regulatory network.

Furthermore, we also observed that SLC7A11-AS1 was predicted to regulate the driver gene CCNE1 by competing for the miRNAs, including hsa-miR-125b-5p, hsa-miR-195-5p, hsa-miR-497-5p, hsa-miR-4524a-5p, and hsa-miR-26b-5p (**Figure 5C**). It should be noted that the shared miRNAs were significantly downregulated, and were inversely correlated with both CCNE1 and SLC7A11-AS1. Based on the analyses, the predicted ceRNA networks provided some evidence about the post-transcriptionally regulatory roles of the ncRNAs in HCC.

### Prognostic significance of the dysregulated lncRNAs

To our knowledge, there were no publicly available HCC circRNA expression datasets with clinical characteristics. Moreover, the associations between miRNAs and HCC prognosis have been extensively studied (52–55). Therefore, we only tested the prognostic significance for both tumorigenesis- and subclass-associated dysregulated lncRNAs based on two approaches. Firstly, we evaluated their associations with overall survival in the TCGA cohort (56). However, only 686 of 1,598 dysregulated lncRNAs were detected in the TCGA gene expression dataset due to different gene annotation versions, 68 of which were significantly associated with the HCC overall survival (*p*-value < 0.05), indicating that these lncRNAs had the potential to predict HCC overall survival. Secondly, to further test the prognostic significance of the dysregulated lncRNAs, especially the undetected lncRNAs in the TCGA cohort, we collected 86 HCC samples from four other independent RNA-seq datasets. Combined with the 28 samples used for ncRNA detection and quantification in our study, 69 of the 114 HCC samples were classified into poor or good prognosis subgroup based on the survival-associated genes by Lee et al. (57) (*Materials and Methods*, FDR < 0.05). Differential expression analysis was thus performed on the lncRNA expression. Finally, 498 dysregulated lncRNAs were identified as differentially expressed lncRNAs between HCC samples with poor and good prognosis (FDR < 0.05 and fold change >2 or < ^1^/_2_).

**Figure 6:**
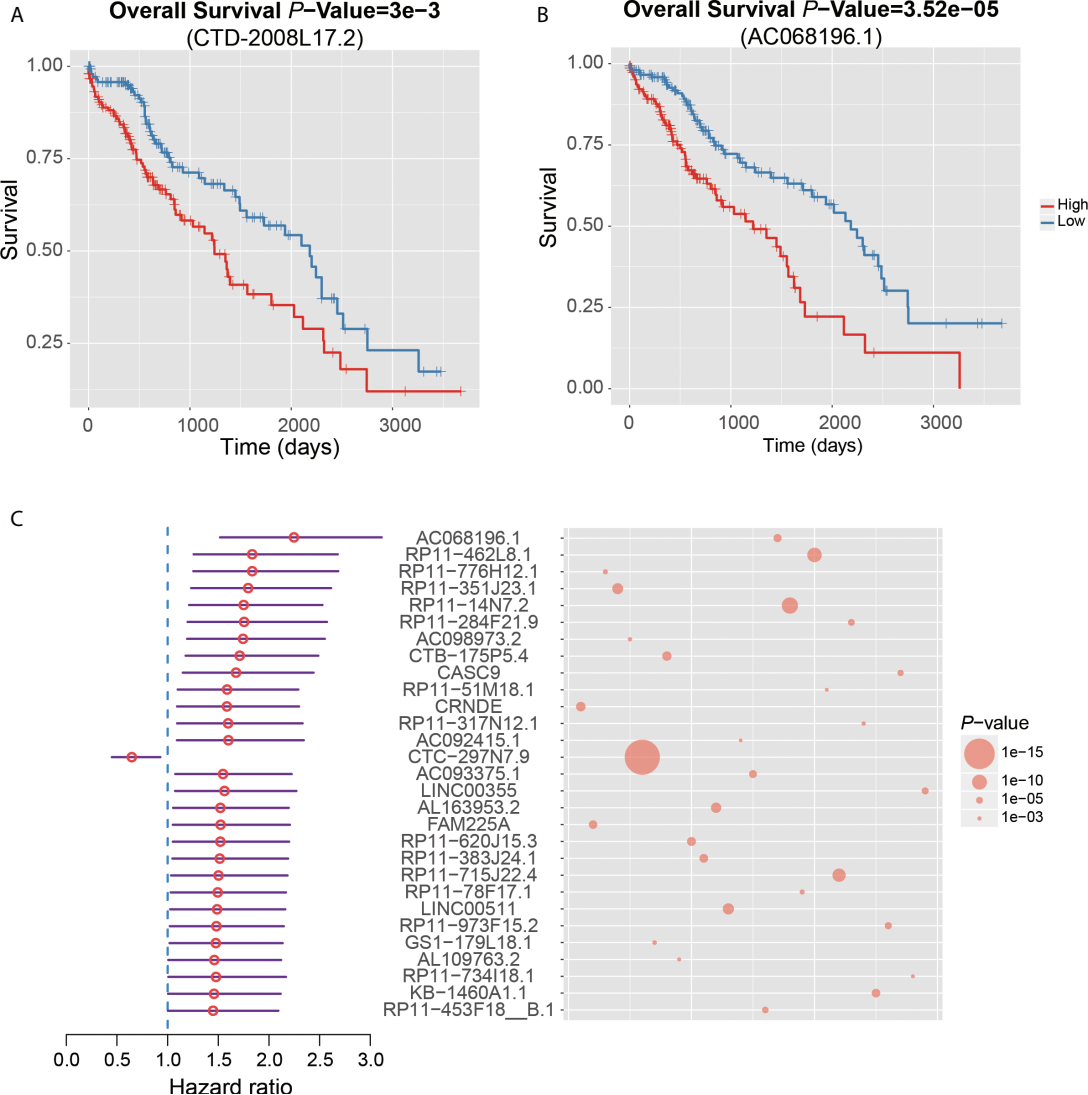
Clinical associations between dysregulated ncRNAs and HCC prognosis. The Kaplan–Meier curves show significant overall survival between HCC samples with high and low expression of CTD-2008L17.2 **(A)** and AC068196.1 **(B)**. **(C)** The 29 dysregulated lncRNAs that are significantly associated with HCC prognosis by both survival analysis of the TCGA dataset and differential expression analysis of HCC samples with good and poor prognosis from six HCC datasets.

Notably, CTD-2008L17.2, which may compete for let-7 family miRNAs with LIN28B, was negatively associated with patient’s survival time in the univariate survival analysis of the TCGA dataset (**Figure 6A**). Twenty-nine lncRNAs were associated with the HCC prognosis based on the two approaches (**Figure 6C**), of which, AC068196.1, upregulated in S2 subclass, was the lncRNA most significantly associated with HCC survival (**Figures 6B, C**). In addition, 313 of the undetected lncRNAs in the TCGA dataset were also closely associated with HCC prognosis based on the comparison of their expression levels between HCC with poor and good prognosis. Interestingly, DUXAP10, the potential HCC diagnostic biomarker in HCC blood exosomes, was also overexpressed in HCC samples with poor prognosis (FDR < 0.05), suggesting that the expression of this lncRNA in exosome may be used to predict patient’s prognosis with validation from further clinical studies. The prognostic analysis based on the two approaches further demonstrated that the functional ncRNAs had the potential to predict patient’s survival.

## Discussion

The molecular basis of HCC about protein-coding genes has largely been studied in the context of tumorigenesis, progression, and metastasis. Despite extensive research about the function of protein-coding genes in HCC, the lack of effective biomarkers for HCC diagnosis or prognostic prediction is still not thoroughly solved. Meanwhile, a majority of non-coding RNAs are characterized to act as cancer driver RNAs, and understanding their deregulation and regulatory roles can facilitate the development of new diagnostic or therapeutic strategies.

To our knowledge, this is the first study utilizing NGS data to simultaneously characterize the dysregulated mRNAs, lncRNA, circRNAs, and miRNAs in HCC and matched para-cancerous tissues. We successfully predicted some novel circRNAs and miRNAs, and quantified their expression levels based on RNA and small RNA sequencing data. The independent dataset validated about ~70% novel circRNAs and miRNAs, and more than 60% dysregulated tumorigenesis-associated ncRNAs, which demonstrated that the data analysis strategies were robust and the predicted novel ncRNAs were highly reliable. Remarkably, the expression levels of circRNA ZRANB1 and lncRNA DUXAP10 that were upregulated in HCC tissues were also observed higher in the blood exosomes of HCC than healthy donors. With the more comprehensive assessment by further experimental and clinical validation, the two ncRNAs may be used for HCC diagnosis.

As the HCC subclasses were well characterized by Hoshida et al., the subclass-associated ncRNAs could be directly linked to HCC clinical characteristics and the activated or inactivated pathways. With the well-characterized clinical phenotypes and dysregulated pathways for the HCC subclasses, we could easily speculate the impact of the dysregulated ncRNAs on HCC tumorigenesis or progression.

Nowadays, more and more studies uncovered the biological function or pathways that some ncRNAs may participate in; however, for most of them, their function still remained unknown. To further uncover the potential regulatory ncRNAs at the post-transcriptional level, we built ncRNA–miRNA–mRNA interaction networks. Non-coding RNAs, including cZRANB1, LINC00501, CTD-2008L17.2, AC092171.4, and SLC7A11-AS1, were identified as functional ncRNAs that competed for the shared miRNAs with mRNAs, and were predicted to be involved in HCC tumorigenesis or progression. In particular, cZRANB1 was upregulated in both HCC tissues and exosomes. The prognostic association analysis highlighted that CTD-2008L17.2, a potential miRNA sponge, and DUXAP10, a potential diagnostic biomarker in HCC exosome, were closely associated with HCC prognosis.

Even though this preliminary study provides an abundant data resource of non-coding RNAs in HCC, the lack of further experimental verification is the major limitation of this study. The systematic analysis revealed that the ncRNAs could function as oncogenic or tumor-suppressive RNAs by regulating gene transcription, post-transcription processes, or other mechanisms, some of which may be used as HCC diagnostic or prognostic biomarkers. Although further characterizing the molecular mechanism of the ncRNAs is extremely essential, our data analysis still provided the hint of how the dysregulated ncRNAs were involved in HCC tumorigenesis or progression for further studies, and novel insights into cancer biology.

## Conclusions

In this study, we identified some novel circRNAs and miRNAs that were validated in the independent dataset. Systematic analysis identified dysregulated ncRNAs that participate in HCC tumorigenesis and progression by inducing transcription of their neighboring genes, increasing their derived miRNAs, or acting as miRNA sponges. Remarkably, two upregulated ncRNAs in the blood exosomes of HCC may be potential diagnostic biomarkers. The ncRNAs competing for miRNAs with mRNAs may be key regulators in HCC, which improved our understanding of the mechanisms about HCC tumorigenesis or progression. Moreover, the prognostic association analysis revealed that the dysregulated ncRNAs with key regulatory roles may also have the potential to predict HCC patients’ prognosis. In summary, our systematic analysis provides not only rich data resources for related researchers, but also new insights into the molecular basis of how different ncRNAs coordinately or antagonistically participate in the pathogenesis process of diseases.

## Materials and methods

### Patient samples

Eight pairs of cancerous and para-cancerous tissues of HCC were obtained from the Department of Hepatopancreatobiliary Surgery (in 2013), the First Affiliated Hospital of Zhejiang University (Hangzhou, China), and were frozen at −70°C.The diagnosis of HCC was confirmed by postsurgery pathology. This project was approved by the Human Research Ethics Committee of the First Affiliated Hospital of Zhejiang University. Prior to surgery, the patients were not treated with anticancer therapy.

All of the eight patients had a history of chronic hepatitis B (CHB), and a single tumor in the liver, without extrahepatic metastasis. Three patients were complicated with alcoholic liver diseases (ALDs), and another three patients had portal vein tumor thrombosis (PVTT) (**Table 1**).

**Table 1:** Clinical information of HCC patients.

Patient ID	Age	Gender	Cirrhosis	ALD	PVTT	Tumor size (cm)	Differentiation	Tumor capsule	BCLC stage	Child score	AFP (ng/ml)
HK08	66	F	No	No	No	2*2	Middle	Complete	A	6	1,007.5
HK09	31	M	Yes	No	Yes	8*8	Middle/poor	Complete	C	5	460.3
XHK019	71	M	No	No	No	4.5*4.8	Middle/well	Complete	A	5	2.8
XHK034	59	M	Yes	Yes	No	10*10	Middle/poor	Complete	A	6	9.3
XHK069	26	F	No	No	Yes	14*8	poor	Incomplete	C	5	50,000
XHK079	60	M	Yes	Yes	No	3.8*3.5	Middle/poor	Incomplete	A	6	13.2
HK1178	55	M	No	No	Yes	8*7	middle	Incomplete	C	5	31,938
XHK096	59	M	Yes	Yes	No	3*2	Middle/poor	Incomplete	A	5	14.4

ALD, alcoholic liver disease; PVTT, portal vein tumor thrombosis; AFP, A-fetoprotein; BCLC, Barcelona Clinic Liver Cancer.

All procedures in the experiment were in agreement with the ethical principles. Patients involved in this study have been informed and signed consent before collection of samples, and the data (which do not involve personal information) related to patients were completely anonymous.

### RNA extraction and sequencing

Total RNAs from HCC and para-cancerous tissues were extracted using TRIzol following the manufacturer’s protocol. For the preparation of RNA-Seq libraries, total RNA was treated with the Ribo-Zero rRNA Removal Kit to remove rRNA according to the manufacturer’s instructions. Standard protocols recommended for small RNA-seq (Illumina TruSeq Small RNA) were used. RNA and small RNA sequencing libraries for the Illumina Hiseq 2000 platform were constructed according to the manufacturer’s instructions (Illumina).

### RNA-seq read mapping, ncRNA detection, and quantification

One hundred-base-pair paired-end reads were mapped to UCSC human reference genome (GRCH37/hg19) using HISAT2 (58) version 2.0.5 with gene models from GENCODE v19 and default options. The SAM format files were compressed and sorted by samtools (59) version 1.3.1. transcripts were quantified by StringTie (60) v1.2.4 at count-based levels. The circRNA was predicted and quantified by CIRI2 (61) based on the alignment by BWA (62). The small RNA-seq data were preprocessed by Trimmomatic (63), and the miRNA prediction and quantification were implemented by miRDeep2 (26). The genes or ncRNAs, which are expressed (read count > 5) in at least 20% of samples, were retained for further analysis. The count-based expression matrix was used for differential expression analysis. The count-based expression values were normalized by a regularized log transformation in DESeq2 (64).

### Differential expression analysis

The count-based expression levels of lncRNAs, mRNAs, circRNAs, and miRNAs were respectively analyzed by DESeq2 (64), which performed differential expression analysis based on negative binomial distribution.

### Pathway enrichment analysis

The enrichment analysis was implemented in GSEA Java software (65). Pathway database was downloaded from MsigDB (http://software.broadinstitute.org/gsea/index.jsp). We only retained canonical pathways curated by KEGG (66) and NCI-PID (67) for enrichment analysis.

### Cox regression-based survival analysis

The survival analysis based on the Cox regression model was implemented in R with packages *survival* and *survcomp.* The comparison of survival curves for high- and low-expression groups was performed using the *G-rho* family of tests with *survdiff* function.

### Collection of independent datasets and identification of prognostic lncRNAs

We collected 106 HCC samples from five independent RNA-seq datasets, with accession numbers SRP039694 (68), SRP050551 (69), SRP062885 (70), SRP068976 (71), and SRP069212 (29) from the SRA (Sequence Read Archive, https://www.ncbi.nlm.nih.gov/sra) database. In particular, the RNA library of the SRP069212 dataset was constructed with the rRNA depletion protocol and small RNA data with accession number SRP068498, which was used to validate the detection and dysregulation of lncRNAs, circRNAs, and miRNAs. In addition, the accession numbers for RNA-seq data of the blood exosomes from HCC and healthy donors were SRP109668 and SRP109666. We adopted the same method to analyze the RNA-seq data of the blood exosomes and detect ncRNAs in blood exosomes.

To identify prognostic lncRNAs, the 106 HCC samples were classified into poor and good prognostic subgroups by the NTP algorithm (72). The survival signatures were obtained from the previous study by Lee et al. (57). The differential expression analysis was conducted between HCC samples with poor and good prognosis to identify the prognostic lncRNAs.

### MiRNA–target prediction

MiRNA binding sites of lncRNAs and circRNAs are predicted by miRanda (73) with strict mode, that is, miRNA–target demands strict 5’ seed pairing. Moreover, the miRNAs and targets should be dysregulated with opposite directions; for example, if the miRNA is upregulated in tumor, its target gene should be downregulated in tumor, and *vice versa*. In addition, the interactions of mRNAs and miRNAs were downloaded from the miRTarBase database (43) (http://mirtarbase.mbc.nctu.edu.tw/php/index.php), which curated the experimentally validated microRNA–target interactions, and TargetScan (42) databases. A ceRNA network was constructed based on the hypergeometric test (*p*-value < 1e-6), the correlation between lncRNA/circRNA and mRNAs (>0.6), and the number of shared miRNAs (>4).

## Data availability statement

The datasets presented in this study can be found in online repositories. The names of the repository/repositories and accession number(s) can be found in the article/**Supplementary Material.**

## Author contributions

In this study, JC conducted the experiment design, XX collected the samples, TS and LZ designed the data analysis, and LZ performed the data analysis. LZ and CW drafted the manuscript. TS, JC, XX, and XL revised and finalized the manuscript. All authors contributed to the article and approved the submitted version.

## Funding

This work was supported by the National High Technology Research and Development Program of China (2012AA020204 and 2015AA020108), the China Human Proteomics Project (2014DFB30010 and 2014DFB30030), the National Science Foundation of China (31671377), the Natural Science Foundation of Shanghai (No. 16ZR1429100), and Shanghai 111 Project (B14019).

## Conflict of interest

The authors declare that the research was conducted in the absence of any commercial or financial relationships that could be construed as a potential conflict of interest.

## Publisher’s note

All claims expressed in this article are solely those of the authors and do not necessarily represent those of their affiliated organizations, or those of the publisher, the editors and the reviewers. Any product that may be evaluated in this article, or claim that may be made by its manufacturer, is not guaranteed or endorsed by the publisher.
